# Supplementary material for: A complete telomere-to-telomere genome assembly of Solanum melongena uncovers key regulators in pan-tissue anthocyanin biosynthesis
Source: Plant Commun. 2025 Sep 23;6(12):101533. doi: 10.1016/j.xplc.2025.101533 (PMC12744757; doi:10.1016/j.xplc.2025.101533)
Supplement: Document S1. Supplemental Figures 1–7 [file mmc1.pdf]

Plant Communications, Volume *6*

Supplemental information

A complete telomere-to-telomere genome assembly of *Solanum melongena* uncovers key regulators in pan-tissue anthocyanin biosynthesis

Qingzhen Wei, Wuhong Wang, Yunzhu Wang, Jiaqi Ai, Tianhua Hu, Haijiao Hu, Jinglei Wang, Yaqin Yan, Hongtao Pang, Na Hu, and Chonglai Bao

Supplemental Figures

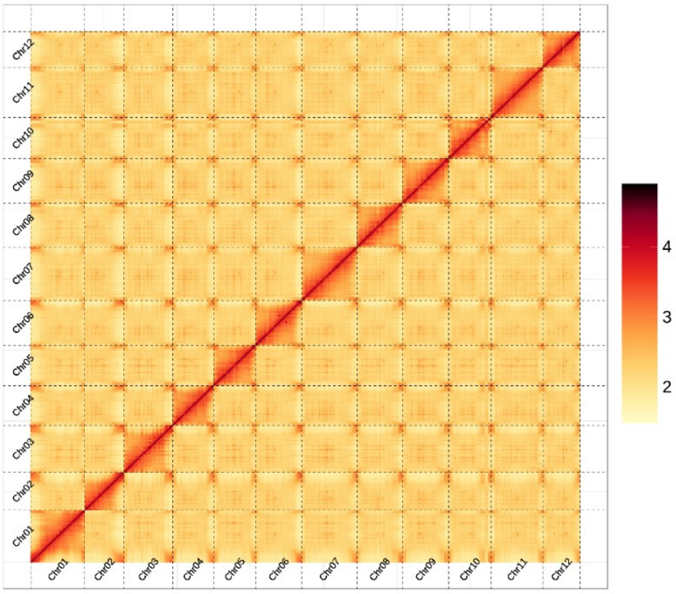

Figure S1 Hi-C chromatin interaction map of the Smel HQ v2.0 assembly.

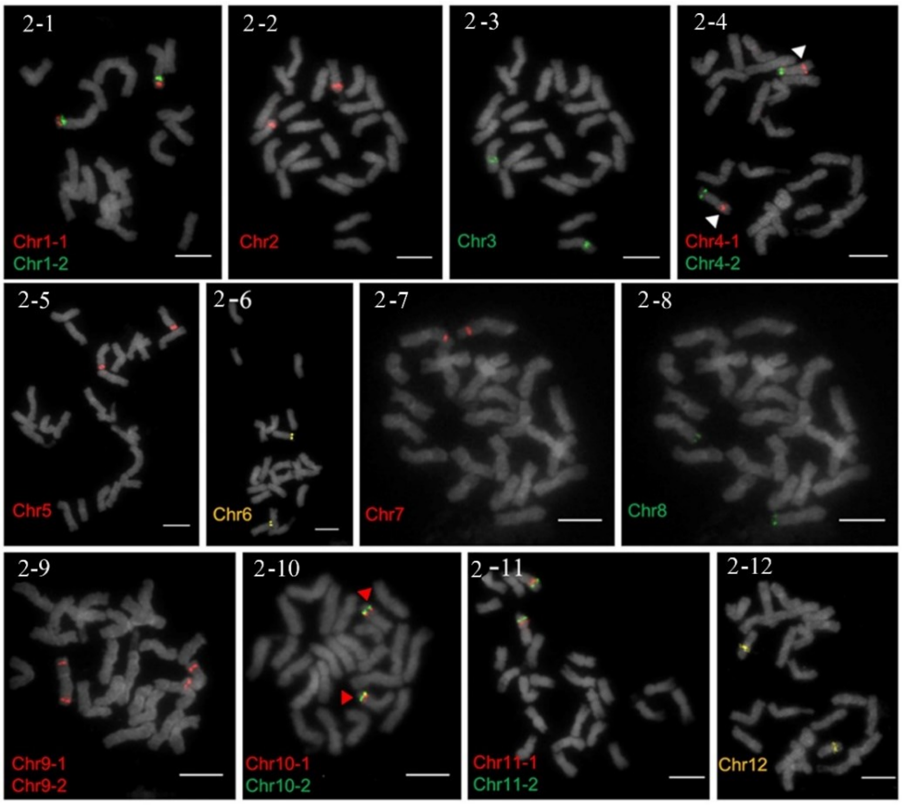

Figure S2 Distribution of the 19 oligo probes on mitosis metaphase chromosomes designed for the identification of 12 eggplant chromosomes and inversion validation. 2-1 to 2-12 showed FISH signals of the oligo probe pools on chromosomes 1-12, respectively.

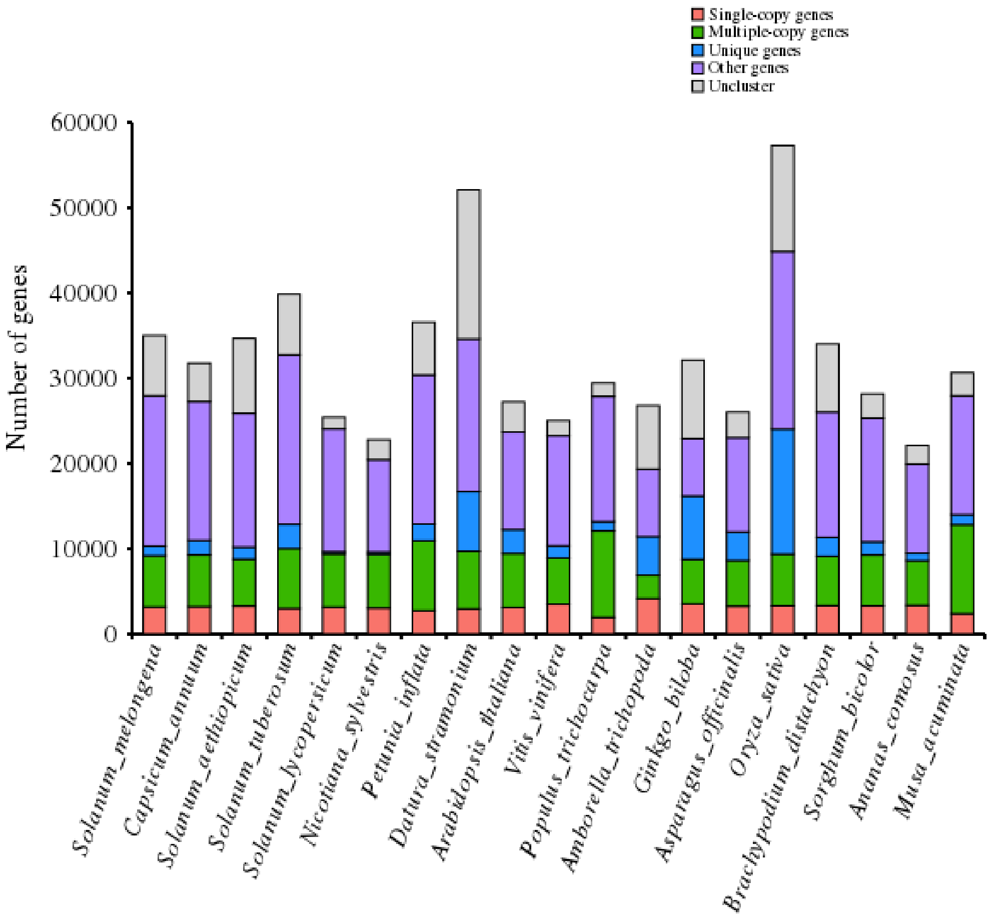

Figure S3 The distribution of genes in different species.

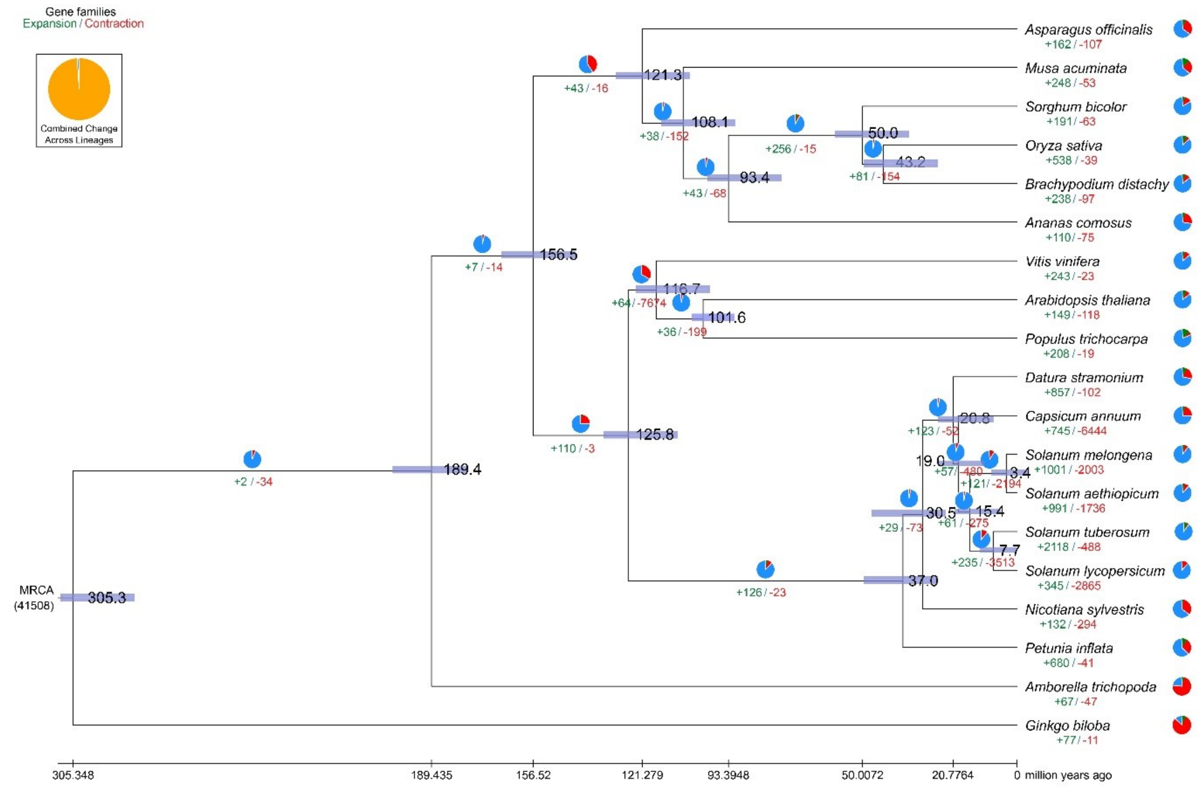

Figure S4 Phylogenetic relationship, divergence time, and expansion and contraction of gene families between S. melongena and 18 plant species. MRCA: most recent common ancestor. The divergence times (in million years) are indicated at each node in blue rectangles. The numbers of expanded gene families are indicated with green numbers, and contracted gene families are indicated with red. represent the conserved gene families are represented by blue portions in the pie charts, while the gene families of MRCAs that expanded or contracted during late divergence are represented by orange proportions.

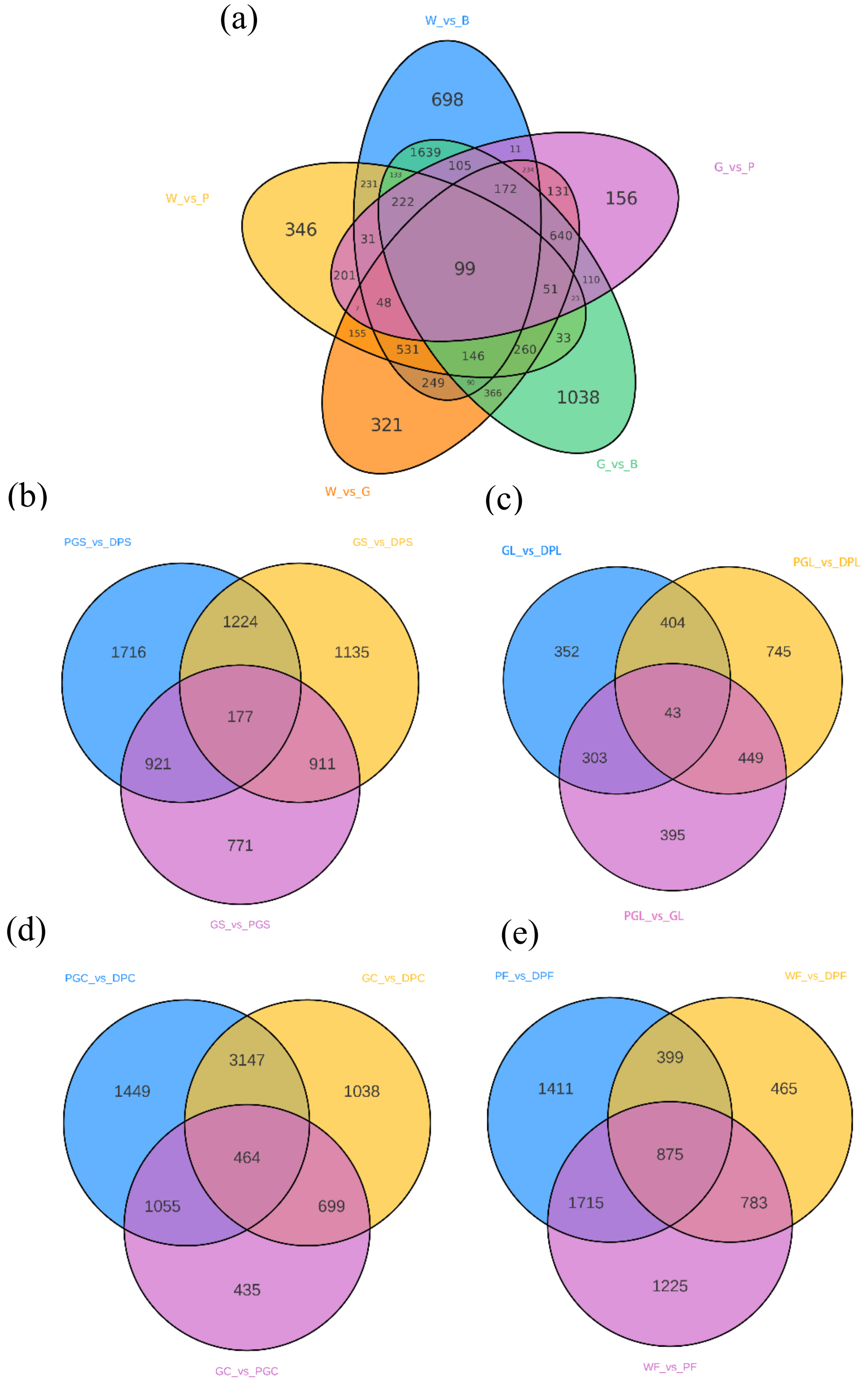

Figure S5 Venn diagram of the common and unique gene in different tissue. (a) peel; (b) stem; (c) young leaf; (d) calyx; (e) flower

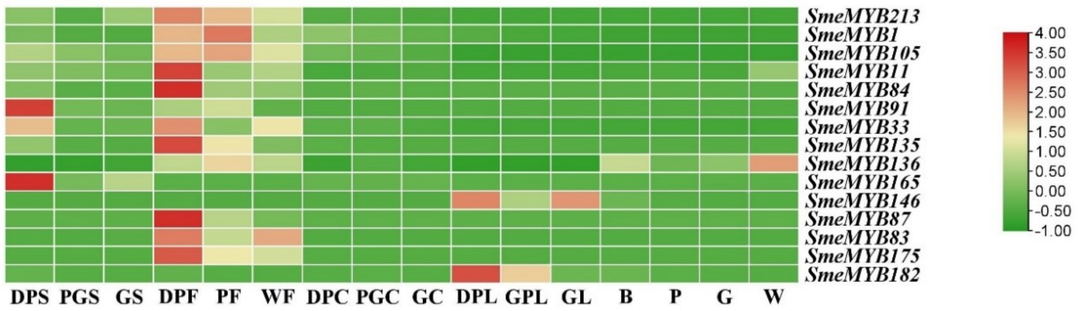

Figure S6 Gene expression of *MYB* transcription factors associated with anthocyanin synthesis in different eggplant tissues. Expression of the *SmeMYBs* in subgroups S4, S5, S6, S7 and S9 that may be associated with anthocyanin synthesis, according to phylogenetic relationships with that of *Arabidopsis*.

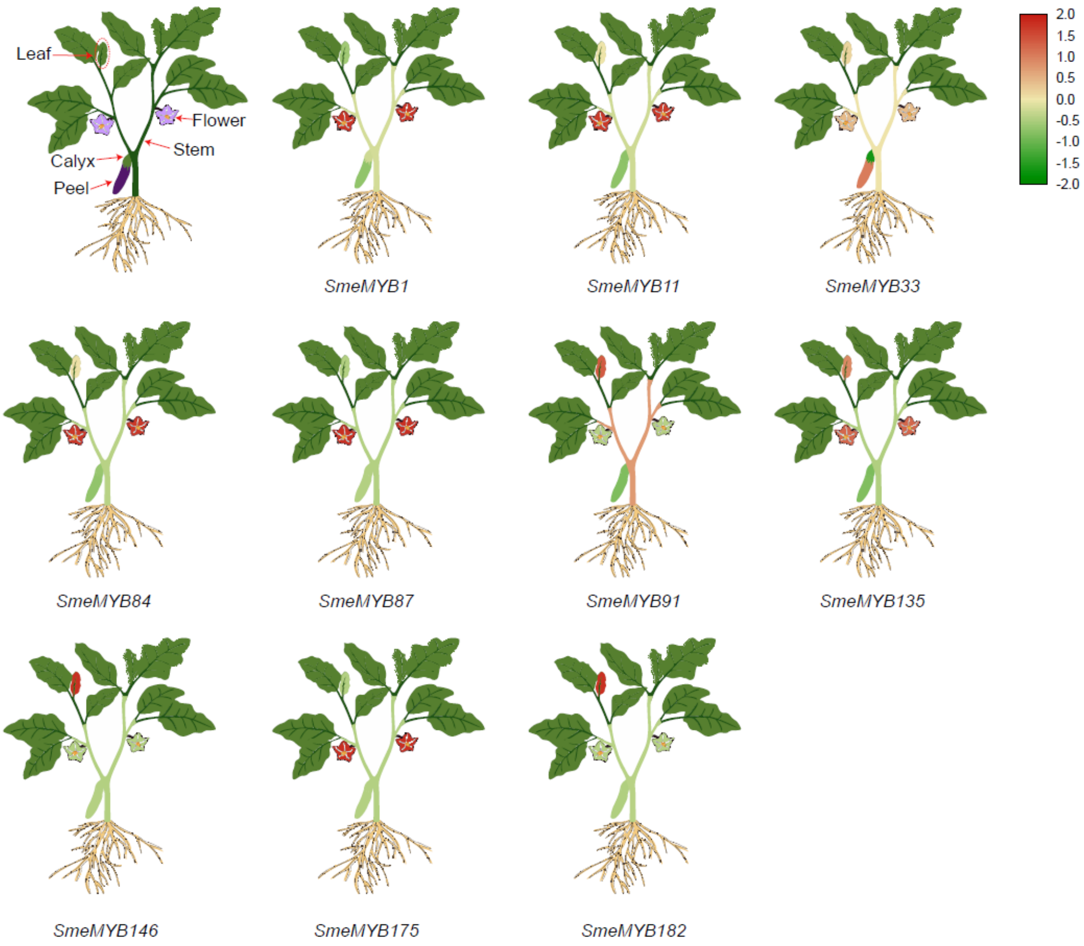

Figure S7 Expression patterns of the 10 differentially expressed *SmeMYBs* in different tissues of HQ-1315. Data were normalized to construct the heatmap. Green indicates low transcript abundance, and red indicates high transcript abundance.
