# Supplementary material for: A complete telomere-to-telomere genome assembly of Solanum melongena uncovers key regulators in pan-tissue anthocyanin biosynthesis
Source: Plant Commun. 2025 Sep 23;6(12):101533. doi: 10.1016/j.xplc.2025.101533 (PMC12744757; doi:10.1016/j.xplc.2025.101533)
Supplement: Document S2. Supplemental methods [file mmc2.pdf]

Supplementary methods

Plant materials

Whole genome sequencing was used to construct a high-quality T2T genome of a high-generation inbred eggplant variety HQ-1315 with fuchsia liner fruit. During the growth period of HQ-1315, the fruit shows fuchsia color, and anthocyanins accumulated in the flowers, stems, leaves, calyx and fruit of the eggplant (the underside of the calyx is white, indicating that anthocyanin synthesis is affected by light). On the other hand, the eggplant 1815 has green flowers, stems, leaves, calyx and fruits during the growth period. 1818 has green fruit peel, whereas 1820 is with white peel during development. Except for these, another 5 materials (1825, 1838, 1873, 1828, 1897) were also used for transcriptome sequencing of the different eggplant tissues. All the eggplant resource materials were cultivated in the greenhouse of Qiaosi Field of the Vegetable Institute of Zhejiang Academy of Agricultural Sciences.

DNA extraction, library construct and genome sequencing

The DNA sequencing process and method were the same as described in previous study (Wei et al., 2020). Briefly, fresh leaves of HQ-1315 were used for high-quality DNA extraction, followed by construction of Illumina library, PacBio HiFi library, ONT (Oxford Nanopore Technologies) library and Hi-C library. All library preparation involves randomly breaking high-quality DNA samples into segments through the Covaris ultrasonic crusher. The Illumina library was sequenced using Illumina NovaSeq PE150. The PacBio HiFi library was sequenced on the PacBio Sequel IIe platform (CLR mode), and then the output raw subreads bam fle was converted to fastq format. In order to estimate the genome size, k-mer analysis was used to estimate the genome size based on Illumina sequencing data. All the DNA extraction, library construction and sequencing procedures were performed according to the manufacturer's protocols.

De novo genome assembling and assessment

Hifiasm (Cheng et al., 2021) was applied to assemble the PacBio Hifi reads. Paired-end clean reads obtained from the Illumina platform were aligned to the eggplant assembly

using BWA software (v0.7.17; Li et al., 2009). Since we are utilizing a high-generation self-crossing sister line derived from the same material as HQ-1315, the Hi-C data directly leverage the previously sequenced datasets (Wei et al., 2020). Based on the Hi-C data obtained by sequencing, the assembled contigs/scaffolds sequence was mounted to near-chromosome level using Allhic (v0.9.8; Zhang et al., 2019), then manually corrected according to the intensity of chromosome interaction using juicebox software. Genome assembly is mainly based on the TGS-GapCloser approach (Xu et al., 2020). BUSCO (Benchmarking Universal Single-Copy Orthologs: http://busco.ezlab.org/) uses Merqury (v1.3; https://github.com/marbl/merqury) to evaluate the integrity of the assembled genome.

Genome annotation

A combined strategy based on homology alignment and *de novo* search to identify the whole genome repeats was applied in our repeat annotation pipeline. Tandem Repeat was extracted using TRF（http://tandem.bu.edu/trf/trf.html）by *ab initio* prediction Wei et al. (2020). The homolog prediction was carried out using Repbase (http://www.girinst.org/repbase) database. RepeatMasker (http://www.repeatmasker.org/) software (Smit et al., 2010) was used to extract repeat regions. A custom library (a combination of Repbase and our *de novo* TE library which was processed by uclust to obtain a non-redundant library) was subjected to RepeatMasker for DNA-level repeat identification. Gene models were annotated by combining *de novo* prediction, homology prediction and RNA-Seq assisted prediction. Homologous protein sequence was found in Ensembl/NCBI/others and then compared with the genome. GeneWise (v2.4.1) (Birney et al., 2004) software was used to accurately splice and compare matching proteins with homologous genome sequences to predict the gene structure contained in each protein region. Gene predication based on *ab initio* was performed as described in Wei et al. (2020) in our automated gene prediction pipeline. PASA (Haas et al., 2003) (Program to Assemble splicing Alignment) was used to merge the genes predicted by the three methods with EvidenceModeler (EVM, v1.1.1) (Haas et al., 2008).

Gene functions were assigned according to the best match by aligning the protein sequences to the Swiss-Prot (Bairoch et al., 2000) with a threshold of E-value ≤ 1e−5. The public databases such as Pfam (Finn et al., 2014), SMRT (Letunic et al., 2004), PANTHER (Mi et al., 2012) and PROSITE (Sigrist et al., 2013) were searched. InterProScan (v5.31) (Mulder et al., 2007) was used to annotate motifs and domains. According to the corresponding InterPro entry assigned the Gene Ontology (GO) IDs (Ashburner et al., 2000). We predicted the proteins function by transferring annotations from the closest BLAST hit (E-value <10-5) in the Swissprot database and BLAST hit (E-value <10-5) in the NR database.

Chromosome preparation and barcode oligo-FISH

Seeds of (eggplant) were treated in moist petri dishes at 25 °C to obtain root tips. Root tips were treated with 0.002M 8-Hydroxyquinoline for 3 h and subsequently fixed in Carnoy's solution. The fixed root tips were digested with an enzyme mixture containing 4% cellulose RS (Yakult), 4% pectinase (Sigma-Aldrich) and 4% pectolase (Yakult) in 0.01M citrate buffer (pH= 4.8), at 37 °C for 40 – 60 min. The procedure of chromosome preparations was performed as described previously (Zhao et al., 2021). The genome of Smel HQ v2.0 was used to generate single-copy 50 nt oligos by Chorus2 software with the parameter "-l 50 -step 5 -homology 80" (Zhang et al., 2021). Oligos were discarded if mapping to two or more locations (with 80% homology) in the genome. The 17 genomic regions on 12 chromosomes were selected for obtaining barcode oligos to distinguish individual chromosomes and verify inversions (Figure 1). A total of 26,239 oligos was used for oligo library synthesis, as described in the published protocols (Bi et al., 2020; Zhao et al., 2021).

The oligo probes were synthesized using fluorescence-labelled primers (Supplemental Table 13) according to published protocols (Bi et al., 2020). The predicted 12 telomeric sequences were marked as probes by labelling fluorophores at the 5' end of sequences. Three types of probes including barcode oligos, telomeric sequences, and rDNA (45s and 5s) were used in FISH experiments. The oligo-FISH protocol was performed as described previously (Zhao et al., 2021). Specifically, the hybridization mixtures

contained 10 μL of 100% Formamide deionized, 2 μL of 2×SSC, 1 μL of oligo probes, 4 μL of 50% dextran sulfate. Denatured mixtures were covered onto prepared slides and then placed at 37 °C for overnight treatment. Chromosomes were stained with 4',6-diamidino-2-phenylindole (DAPI) in VectaShield antifade solution (Vector Laboratories). The FISH signals were acquired separately in different channels using a SENSYS CCD camera attached to an Olympus BX51 microscope. The FISH images were further processed through Adobe Photoshop software.

Gene family clustering and phylogenetic analysis

Gene sets from each species underwent sequential filtering. First, for genes possessing multiple transcript variants (alternative splicing), only the transcript encoding the longest coding sequence (CDS) was retained. Second, genes encoding proteins shorter than 50 amino acids, or containing stop codons, were filtered out. Protein sequence similarity among all species were determined using an all-vs-all BLASTP search, employing a default E-value cutoff of 1×10^{-5}. The resulting data was subsequently clustered using OrthoMCL software (http://orthomcl.org/orthomcl/) to identify orthologous gene families. The OrthoMCL analysis was performed using an inflation index parameter of 1.5. 19 plant species were used in gene family clustering analysis, resulting in the identification of 41,536 orthologous gene families. Among these, 559 gene families represented common single-copy orthologs present in all species (Supplemental Figure 3).

All single-copy gene families identified above were individually aligned using MUSCLE (v3.8.31, http://www.drive5.com/muscle/). Subsequently, the resulting individual alignments were concatenated into a single super alignment matrix. Finally, a maximum likelihood (ML) phylogenetic tree (Guindon et al., 2010) was constructed from the super alignment matrix using RAxML (v8.2.12, http://sco.hits.org/exelixis/web/software/ raxml/index.html).

Divergence Time Estimation

Divergence times among species were estimated using the set of 559 single-copy orthologous gene families. All time units are in millions years ago (Ma). The analysis was performed using the mcmctree program within the PAML software package (v4.9,

http://abacus.gene.ucl.ac.uk/software/paml. html). Fossil calibration points were applied to constrain specific nodes within the phylogeny: *Oryza sativa* - *Brachypodium distachyon* (42–52 Ma), *Sorghum bicolor* - *Oryza sativa* (40–52 Ma), *Asparagus officinalis* - *Musa acuminata* (104–125 Ma), *Arabidopsis thaliana* - *Populus trichocarpa* (97–109 Ma), *Vitis vinifera* - *Populus trichocarpa* (107–135 Ma), *Capsicum annuum* - *Solanum melongena* (16.1–22.7 Ma), *Solanum tuberosum* - *Petunia inflata* (28–41 Ma), *Arabidopsis thaliana* - *Solanum tuberosum* (111–131 Ma), *Oryza sativa* - *Solanum aethiopicum* (148–173 Ma), *Solanum lycopersicum* - *Amborella trichopoda* (173–199 Ma), *Arabidopsis thaliana* - *Ginkgo biloba* (289–330 Ma). All calibration points were obtained from the TimeTree database (http://www.timetree.org/).

Gene Family Expansion and Contraction analysis

Gene families exhibiting abnormal size distributions across species were filtered prior to analysis. Specifically, gene families where the gene count in one species was ≥ 200, while simultaneously being ≤ 2 in all other species, were excluded. Subsequently, gene family expansion and contraction events were inferred across the phylogeny using CAFE software (v5.0, http://sourceforge.net/projects/cafehahnlab/) based on the estimated species divergence times and gene family clustering results.

MYB gene family analysis

A total of 197 *Arabidopsis* MYB protein sequences were used to construct a hidden Markov model (HMM) using HMMER3.0 software. Using the above model, all the coding protein sequences of eggplant were searched, and all potential MYB family sequences in eggplant protein sequences were identified. The sequences on the alignment were used as all potential MYB family sequences. Candidate sequences were obtained using Pfam (v33.1; Finn et al., 2014). A database structure of the target domain annotation, determine contains PF00249, PF11831, PF13921, PF14379 sequence as the final sequence of MYB domain structure. MAFFT (v7.427) was used to perform multiple sequence alignment of the identified eggplant *MYB* family members with the MYBs of *Arabidopsis*, tomato, pepper and potato. MEGA (MEGA10) (Kumar et al., 2008) software was employed to construct the phylogenetic tree using Poisson model,

with the cutoff value of 50%, and the Bootstrap value of 1000. The phylogenetic tree was further analyzed using iTOL v6 (https://itol.embl.de/). Collinearity analysis was performed using MCScanX software with default parameters (MATCH_SCORE: 50, MATCH_SIZE: 5, GAP_PENALTY: -1, OVERLAP_WINDOW: 5, E_VALUE: 1e-05, MAX GAPS: 25). Ka/Ks analysis of gene families was performed using Kaks Calculator (v2.0) software.

Transcriptome sequencing

The peels of 1825 (black-purple, B), HQ-1315 (purple, P), 1815 (green, G) and 1820 (white, W) inbred lines were used for transcriptome sequencing. The flowers of HQ-1315 (dark purple, DPF), 1873 (purple, PF) and 1828 (white, WF) inbred lines were used for RNA sequencing (RNA-seq), and only blooming petals on that day were selected. The calyxes (excluding pulp) of 1825 (dark purple, DPC), 1838 (purple green, PGC), and 1818 (green, GC) were used for RNA-seq. The stems of 1825 (dark purple, DPS), 1818 (purple-green, PGS), and 1828 (green, GS) were used for RNA-seq. The young leaves of 1897 (dark-purple, DPL), 1838 (purple-green, PGL), and 1815 (green, GL) were used for RNA-seq. In addition, the flowers (DPF), stems (with the purple color between DPS and PGS), calyxes (with the purple color between DPC and PGC), leaf young leaves (with the purple color between DPL and PGL), and fruit peels (P) of the eggplant inbred line HQ-1315 were also collected for RNA-seq. Three biological replicates were performed for each sample. Total RNA was extracted from different colored peels, calyxes, flowers and stems using TRIzol reagent kits (Invitrogen, CA, USA) according to the manufacturer's instructions. The final cDNA library was sequenced on Illumina HiSeq platform after achieving the requirements. HISAT2 was used to align the reads to the reference genome (Kim et al., 2015). Differential expression analysis between sample groups was performed using DESeq2 (McKenna et al., 2010; Wang et al., 2014) to identify the set of differentially expressed genes (DEGs). Following the differential analysis, the P-values obtained from the hypothesis tests were adjusted for multiple testing using the Benjamini-Hochberg method to control the False Discovery Rate (FDR). DEGs were selected based on the criteria of

|log2FoldChange| >= 1 and an FDR-adjusted P-value (FDR) < 0.05. Expression quantification was performed using the featureCounts software (Liao et al., 2014), with FPKM (Fragments Per Kilobase of transcript per Million fragments mapped) adopted as the metric to evaluate the expression levels of transcripts or genes. The mean FPKM values of three biological replicates of the *SmeMYB* DEGs in various tissues with different colors were determined. TBtools was utilized to generate a heat map illustrating theDEGs of the *SmeMYB* in various tissues with different colors.

References

Ashburner, M., Ball, C.A., Blake, J.A., Botstein, D., Butler, H., Cherry, J.M., Davis, A.P. et al. (2000) Gene ontology: tool for the unification of biology. The Gene Ontology Consortium. Nat. Genet. **25**: 25–29.

Bairoch, A. and Apweiler, R. (2000) The SWISS-PROT protein sequence database and its supplement TrEMBL in 2000. Nucleic Acids Res. **28**: 45–48.

Bi, Y.F., Zhao, Q.Z., Yan, W.K., Li, M.X., Liu, Y.X., Cheng, C.Y., Zhang, L. et al. (2020) Flexible chromosome painting based on multiplex PCR of oligonucleotides and its application for comparative chromosome analyses in Cucumis. The Plant Journal, **102**: 178–186.

Birney, E., Clamp, M., Durbin, R. (2004) GeneWise and Genomewise. Genome Res. **14**: 988-995.

Cheng, H.Y., Concepcion, G.T., Feng, X.W., Zhang, H.W. Li, H. (2021) Haplotype-resolved *de novo* assembly using phased assembly graphs with hifiasm. Nature Methods. **18**: 1–6.

Finn, R.D., Bateman, A., Clements, J., Coggill, P., Eberhardt R.Y., Eddy, S.R., Heger, A. et al. (2014) Pfam: the protein families database. Nucleic Acids Research **42**: 222–30.

Guindon, S., Dufayard, J.F., Lefort, V., Anisimova, M., Hordijk, W., Gascuel, O. (2010) New Algorithms and Methods to Estimate Maximum-Likelihood Phylogenies: Assessing the Performance of PhyML 3.0. Syst Biol. **59**: 307-321.

Haas, B.J., Delcher, A.L., Mount, S.M., Wortman, J.R., Smith, R.K., Jr, Hannick L.I., Maiti, R. et al. (2003) Improving the *Arabidopsis* genome annotation using maximal transcript alignment assemblies. Nucleic Acids Res. **31**: 5654–5666.

Haas, B.J., Salzberg, S.L., Zhu, W., Pertea, M., Allen, J.E., Orvis, J., White, O., et al. (2008) Automated eukaryotic gene structure annotation using EVidenceModeler and the Program to Assemble Spliced Alignments. Genome Biol. **9**: R7.

Kim, D., Langmead, B., Salzberg, S.L. (2015) HISAT: A fast spliced aligner with low memory requirements. Nature Methods. **12**: 357–360.

Kumar, S., Nei, M., Dudley, J., Tamura, K. (2008) MEGA: A biologist-centric software for evolutionary analysis of DNA and protein sequences. Briefings in Bioinformatics. **9**: 299–306.

Letunic, I., Couple, R.R., Schmidt, S., Ciccarelli, F.D., Doerks, T., Schultz, J., Ponting, C.P. et al. (2004) SMART 4.0: towards genomic data integration. Nucleic Acids Res. **32**: 142–144.

Li, H. and Durbin, R. (2009) Fast and accurate short read alignment with Burrows–Wheeler transform. Bioinformatics. **25**: 1754–1760.

Liao, Y., Smyth, G.K., Shi, W. (2014) FeatureCounts: An efficient general purpose program for assigning sequence reads to genomic features. Bioinformatics (Oxford, England). **30**: 923–930.

McKenna, A., Hanna, M., Banks, E., Sivachenko, A., Cibulskis, K., Kernytsky, A., Garimella, K. et al. (2010) The genome analysis toolkit: A mapreduce framework for analyzing next-generation DNA sequencing data. Genome Res. **20**: 1297-303.

Mi, H. Y., Muruganujan, A., Thomas, P. D. (2012) PANTHER in 2013: modeling the evolution of gene function, and other gene attributes, in the context of phylogenetic trees. Nucleic Acids Res. **41**: 377–386.

Mulder, N. and Apweiler, R. (2007) InterPro and InterProScan: tools for protein sequence classification and comparison. Methods Mol Biol. **396**: 59–70.

Sigrist, C. J. A., Castro, E.D., Cerutti, L., Cuche, B.A., Hulo, N., Bridge, A., Bougueleret, L. et al. (2013) New and continuing developments at PROSITE. Nucleic Acids Res. **41**: 344–347.

Smit, A.F.A., Hubley, R., Green, P. (2010) RepeatMasker Open-3.0 (Seattle: The Institute for Systems Biology).

Wang, K., Li, M., Hakonarson, H. (2010) ANNOVAR: Functional annotation of genetic variants from high-throughput sequencing data. Nucleic Acids Res. **38**: e164.

Wei, Q.Z., Wang, J.L., Wang, W.H., Hu, T.H., Hu, H.J., Bao, C.L. (2020) A high-quality chromosome-level genome assembly reveals genetics for important traits in eggplant. Hortic Res. **7**: 153.

Xu, M.Y., Guo, L.D., Gu, S.Q., Wang, O., Zhang, R., Peters, B.A., Fan, G.Y. et al. (2020) TGS-GapCloser: A fast and accurate gap closer for large genomes with low coverage of error-prone long reads. Giga Sci. 9: giaa094.

Zhang, T., Liu, G.Q., Zhao, H.N., Braz, G.T., Jiang, J.M. (2021) Chorus2: design of genome-scale oligonucleotide-based probes for fluorescence in situ hybridization. Plant Biotechnol J. **19**: 1967-1978.

Zhang, X.T., Zhang, S.C., Zhao, Q., Ming, R., Tang, H.B. (2019) Assembly of allele-aware, chromosomal-scale autopolyploid genomes based on Hi-C data. Nat Pla nts. **5**: 833-845.

Zhao, Q.Z., Meng Y., Wang P.Q., Qin X.D., Cheng, C.Y., Zhou, J.G., Yu, X.Q. et al. (2021) Reconstruction of ancestral karyotype illuminates chromosome evolution in the genus Cucumis. The Plant Journal. **107:** 1243–1259.
