# Supplementary material for: A complete telomere-to-telomere genome assembly of Solanum melongena uncovers key regulators in pan-tissue anthocyanin biosynthesis
Source: Plant Commun. 2025 Sep 23;6(12):101533. doi: 10.1016/j.xplc.2025.101533 (PMC12744757; doi:10.1016/j.xplc.2025.101533)
Supplement: Document S3. Article plus supplemental information [file mmc4.pdf]

Plant Communications
Correspondence

CellPress
Partner Journal

A complete telomere-to-telomere genome assembly of *Solanum melongena* uncovers key regulators in pan-tissue anthocyanin biosynthesis

Dear Editor,

Eggplant (*Solanum melongena*) belongs to the genus *Solanum* (Solanaceae), which ranks third in global production among Solanaceae crops. Although four cultivated eggplant genomes (Hirakawa et al., 2014; Barchi et al., 2019, 2021; Wei et al., 2020; Li et al., 2021) and two wild eggplant genomes (Song et al., 2019; Zhang et al., 2023) had been published by 2024, numerous gaps and unassembled regions remained, including telomeres and centromeres. Recently, Fang et al. (2025) reported a telomere-to-telomere (T2T) eggplant genome (Smel NO211) with nine gapless chromosomes and 22 telomeres from a green-striped cultivar. However, there are still nine gaps remaining in the Smel NO211 assembly. Moreover, the 12 pseudo chromosomes of eggplant have not been verified or anchored to cytogenetic chromosomes. Filling these gaps and generating a more contiguous, complete genome sequence is crucial for gene mining, functional studies, marker-assisted breeding, and evolutionary biology.

In this study, we assembled a gap-free T2T genome of the linear eggplant inbred line "HQ-1315" using multiple platforms, including Oxford Nanopore Technologies (ONT), PacBio HiFi, and Hi-C (Figure 1A; Supplemental Figure 1). The genome size was estimated to be 1208.95 Mb based on 57.20 Gb of Illumina reads. The heterozygosity rate of the genome was approximately 0.13% (Supplemental Table 1). Contigs were initially assembled using 51.36 Gb of PacBio HiFi and 51.10 Gb of ONT reads (Supplemental Tables 2 and 3), generating a draft genome of 1160.22 Mb (Supplemental Table 4). Subsequently, 131.73 Gb of Hi-C clean reads were used to construct physical maps by ordering and clustering scaffolds into 12 chromosome-level pseudomolecules. ONT reads were then aligned to fill the remaining 40 gaps, closing 36 of them. The final four gaps were resolved using the newly published Smel NO211 reference genome (Fang et al., 2025), achieving a complete, gap-free T2T assembly. The 12 pseudo chromosomes were obtained with a total length of 1161.12 Mb, and the total sequence length anchored to the chromosomes was 1138.66 Mb (98.06%). The contig N50 reached 53.47 Mb. BUSCO analysis (99.50% completeness) and the consensus quality value (43.67; 99.99% completeness) confirmed the high quality of Smel HQ v.2.0 (Supplemental Table 5). QuarTeT software predicted 12 centromere locations (Figure 1B; Supplemental Table 6). Repetitive sequence annotation showed that the Smel HQ v.2.0 eggplant genome contained 811.89 Mb (69.92%) of repeats, most of which were long terminal repeats (65.30% of the assembled sequences; Supplemental Table 7). Chromosomes were numbered consistently with previous eggplant genomes (Wei et al., 2020; Barchi et al., 2021; Li et al., 2021). Notably, the contig N50 of Smel HQ v.2.0 increased nearly tenfold—from 5.26 Mb in Smel HQ v.1.0 and 5.3 Mb in Smel GQ to 53.46 Mb. Compared with Smel NO211 (Fang et al., 2025), Smel HQ v.2.0 assembled 12 gap-free chromosomes and achieved a higher genome anchoring rate (98.06% vs. 95.28%), suggesting a more complete T2T assembly. Because annotation data for Smel NO211 were not available, further comparisons could not be performed. Overall, Smel HQ v.2.0 excels in completeness and annotation.

A consensus cytogenetic and physical map of the Smel HQ v.2.0 T2T eggplant genome was constructed using oligonucleotide-fluorescence *in situ* hybridization (oligo-FISH). Sixteen regions (0.5–3 Mb) across the 12 chromosomes were selected, from which 24 000 oligo probes were designed, with an average pool size of 1500 oligos. A mitotic chromosome karyotype of eggplant was constructed and successfully integrated with the 12 chromosomes of the Smel HQ v.2.0 assembly (Figure 1B and 1C). Gene distribution analysis revealed relatively high density at the terminal regions of the chromosomes. Telomeric tandem repeats were predicted with Telomeric-identifier (v.0.2.0), showing AACCCTG/CAGGGTT repeats in eggplant telomeres, which were further confirmed by FISH (Figure 1D, d-1 and d-2; Supplemental Table 8). Nonetheless, differences in FISH signal strength indicated uneven distribution among chromosomes and significant variation in repeat number. In addition, 45S and 5S ribosomal DNA (rDNA) loci were identified using FISH. The results show one pair of 45S rDNA loci on the short arm of chromosome 2 (chr2) and one pair of 5S rDNA loci on the long arm of chr5 (Figure 1D, d-3–d-5). We compared the three genome assemblies—Smel HQ v.1.0 (Wei et al., 2020), Smel 67/3 v.4.1 (Barchi et al., 2021), and Smel HQ v.2.0—and identified inversions and gaps in several chromosomes among them (Figure 1E). To verify the quality of the three genomes, oligo probe pools were designed from four chromosomes of Smel HQ v.2.0. Two pairs of oligo probe pools (chr1-1/2 and chr11-1/2) were designed from both sides of the predicted inversions on chr1 and chr11 between Smel HQ v.2.0 and Smel 67/3 v.4.1 (Figures 1E and 1F). The locations of FISH signals were distributed as designed, and no inversions occurred, indicating accurate sequence order on Smel HQ v.2.0 chr1 and chr11 (Figure 1F; Supplemental Figure 2). Likewise, the misassembled inversion on chr10 of Smel HQ v.1.0 was corrected in Smel HQ v.2.0, as verified using the chr10-1 and chr10-2 probe pools. Moreover, we found a gap within the 22–25 Mb region on chr4 of Smel HQ v.1.0 and Smel 67/3 v.4.1 (Figure 1E, black arrow). Using the chr4-1 oligo probe pool designed from Smel HQ v.2.0, we confirmed closure of this gap, indicating that the Smel HQ v.2.0 assembly shows a significant improvement in gap filling. In conclusion, three inversion corrections and one

This is an open access article under the CC BY license (http://creativecommons.org/licenses/by/4.0/).

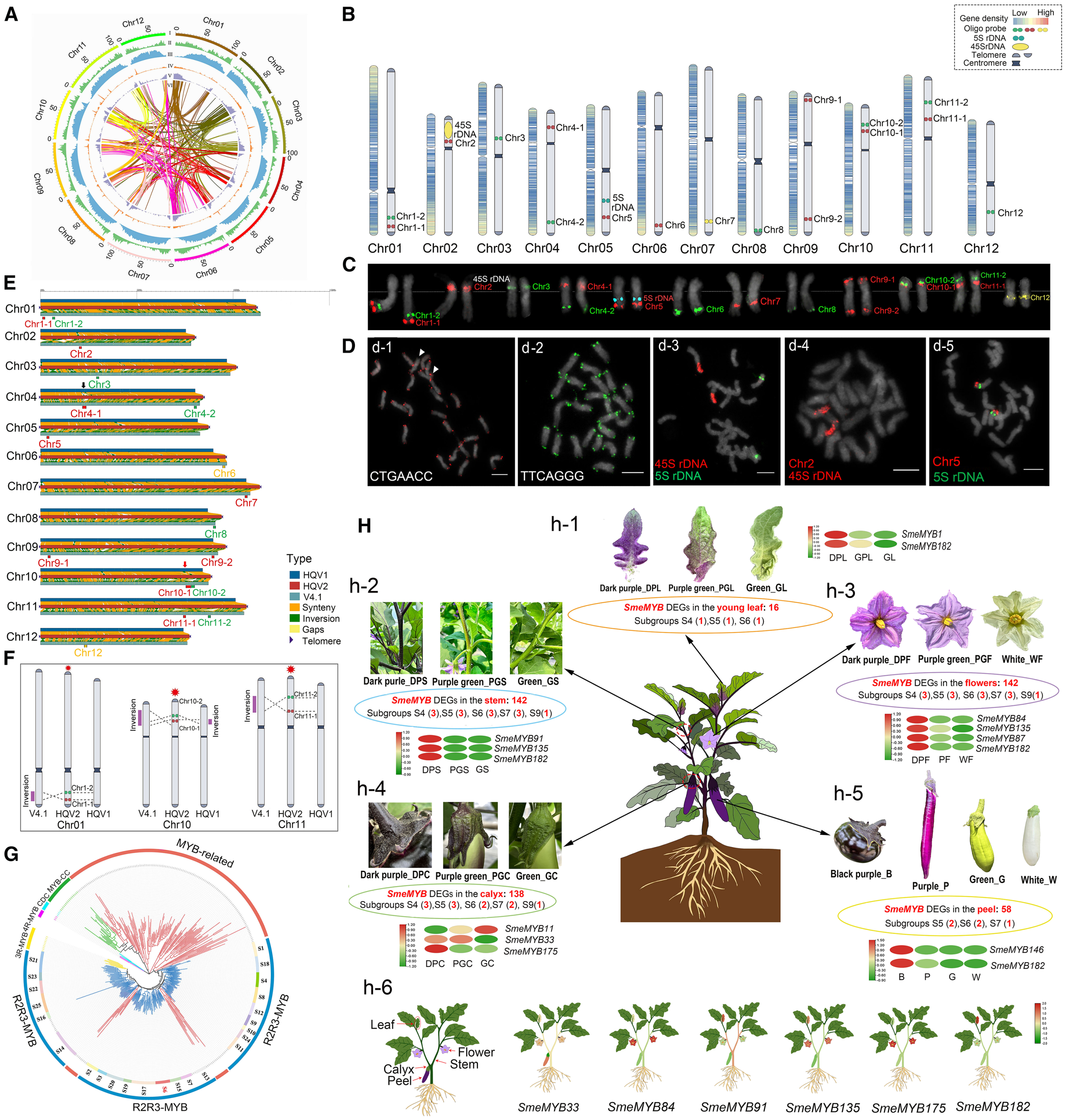

Figure 1. Telomere-to-telomere assembly of the eggplant genome.

(A) Chromosome characterization of the Smel HQ v.2.0 genome. **(I)** Chromosomes. **(II)** Gene density. **(III)** Genome GC content. **(IV)** Repeat coverage. **(V)** Syntenic blocks.

(B) Ideogram of the eggplant chromosome karyotype. The left side shows gene density distribution along chromosomes and the relative positions of centromeres. The right side shows the eggplant karyotype diagram, indicating the relative positions of telomeres, 5S and 45S rDNA loci, and the distribution of 17 specific oligo-probe pools on 12 eggplant chromosomes.

(C) Karyotype of mitotic metaphase chromosomes that unambiguously distinguishes the 12 chromosomes of the eggplant cultivar HQ-1315.

(D) Distribution of telomeric sequences and 5S and 45S rDNA in eggplant. **(d-1)** Chromosomal distribution of CTGAACC telomeric sequences. **(d-2)** Chromosomal distribution of TTCAGGG telomeric sequences. **(d-3)** Chromosomal distribution of 5S and 45S rDNA. **(d-4)** Co-localization of 45S rDNA and the chromosome-specific oligo pool on chromosome 2. **(d-5)** Co-localization of 5S rDNA and the chromosome-specific oligo pool on chromosome 5. Scale bars, 5 μm.

(legend continued on next page)

gap closure were verified, indicating a much-improved assembly quality for the Smel HQ v.2.0 genome.

A total of 35 004 coding genes were predicted, and 97.66% of these genes were annotated in at least one database (Supplemental Tables 9 and 10). In addition, 4012 transfer RNA genes, 7713 ribosomal RNA genes, 562 small nuclear RNA genes, and 297 microRNA genes were predicted (Supplemental Table 11). A phylogenetic tree comprising *S. melongena* (Smel HQ v.2.0) and 18 other plant species was constructed, and divergence times were estimated based on the genes from 559 single-copy families (Supplemental Figures 3 and 4). As expected, *S. melongena* clustered with *S. aethiopicum* within the Solanaceae clade, which diverged approximately 7.7 million years ago. Moreover, the expansion and contraction of orthologous gene families were investigated. We found that 61 gene families were expanded in the lineage leading to *Solanum*, whereas 275 gene families were contracted (Supplemental Figure 4). MYB proteins are among the most prevalent transcription factors, playing important roles in regulating plant growth and development, stress resistance, and secondary metabolism, especially in anthocyanin biosynthesis (Jiang and Rao, 2020). Eggplant cultivars and closely related wild species exhibit abundant diversity in color pigmentation, with anthocyanin as the main determinant of the purple color (Li et al., 2023). We performed genome-wide identification of MYB proteins in the Smel HQ v.2.0 genome and found 213 MYB members (SmeMYB), including 104 R2R3-MYB proteins, 104 1R-MYB proteins, and 5 3R-MYB proteins (Figure 1G; Supplemental Table 12). An overview of *SmeMYB* expression profiles across eggplant tissues of different colors was obtained by transcriptome sequencing (Figure 1H). A total of 8696, 23 320, 23 362, and 24 055 differentially expressed genes (DEGs) were identified in the peel, calyx, stem, and flower, respectively (Supplemental Figure 5). Among the 213 *SmeMYB*s, 142, 142, 138, 58, and 16 *SmeMYB* DEGs were expressed in the stem, flower, calyx, peel, and leaf, respectively (Supplemental Table 13). Eight *SmeMYB* DEGs were expressed in all five tissues, including five 1R-MYBs, two R2R3-MYBs, and one 3R-MYB. The *SmeMYB182* gene had significantly higher expression levels in the dark purple peel, leaf, stem, and flower than in green or white tissues, whereas the closely related gene *SmeMYB175* was highly expressed in the dark purple calyx. Expression profiles of 15 *SmeMYB*s from the S4–S7 and S9 subfamilies showed that *SmeMYB165* and *SmeMYB91* exhibited high expression levels in B stems, which may also participate in anthocyanin accumulation (Supplemental Figure 6; Dubos et al., 2010). The expression patterns of 10 differentially expressed *SmeMYB*s potentially associated with anthocyanin synthesis in different tissues of the eggplant inbred line HQ-1315 were further investigated (Supplemental Figure 7; Supplemental Table 14). The results showed that six *SmeMYB*s were predominantly expressed in flowers, whereas three *SmeMYB*s had high expression in the leaf (Figure 1H, h-6). *SmeMYB33* showed broad-spectrum expression in the flower, stem, leaf, and peel (Figure 1H, h-6). These results provide a comprehensive understanding of anthocyanin accumulation in eggplant and facilitate time-efficient breeding practices in the future.

We established a comprehensive genomic database for eggplant (http://47.92.172.28:12068/Eggplant/home/index), encompassing the genomic data of cultivated lines Smel NS, Smel 67/3 v.4.1, Smel GQ, Smel HQ v.1.0, and Smel HQ v.2.0, along with those of wild species *S. aethiopicum* and *S. torvum*. The database includes tools for gene function search, sequence alignment, collinearity analysis, and genomic data download. It also provides functions for sequence extraction and primer design. The eggplant database is open access and free of charge, providing a comprehensive platform for eggplant genetic and genomic research.

In summary, we present the first integrated cytogenetic and gap-free T2T genome assembly of eggplant (Smel HQ v.2.0), which will substantially facilitate more in-depth genetic and genomic studies, and shed light on the universal and specific roles of *SmeMYB*s in anthocyanin biosynthesis across tissues.

DATA AVAILABILITY

All data supporting the conclusions of this study are included in the paper and its supplemental information. The assembly and annotation of the T2T genome Smel HQ v.2.0 are openly available at our database: http://47.92.172.28:12068/Eggplant/home/index. Additional data related to this work are available from the first or corresponding author upon reasonable request.

FUNDING

This research was supported by the Youth Project of the National Natural Science Foundation of China (grant no. 32002055) and the New Variety Breeding Project of the Major Science and Technology Projects of Zhejiang (grant no. 2021C02065-1-3).

ACKNOWLEDGMENTS

We thank Dr. Qinzheng Zhao from Nanjing Agricultural University for constructive advice and technical support on oligo probe design and FISH methodology. The authors declare no conflict of interest.

(E) Chromosome collinearity among HQ v.2.0, HQ v.1.0, and 67/3. The collinearity of the 12 chromosomes is shown on the left; three inversions were identified on chromosomes 1, 10, and 11, respectively; the magnified image is located at the top right.

(F) Ideogram showing the relative positions of oligo-probe pools designed for FISH validation of the three inversions.

(G) Phylogenetic tree of 410 MYB protein sequences from eggplant and *Arabidopsis* constructed using the maximum likelihood (ML) method in MEGAX with 1000 bootstrap replicates. MYB proteins are indicated with red (1R-MYBs), blue (R2R3-MYBs), yellow (3R-MYBs), purple (4R-MYBs), green (MYB-CC), and indigo (MYB-CDC) branches and arcs. R2R3-MYBs are grouped into 25 subfamilies (S1–S25), indicated with different colored arcs.

(H) Overview of the divergent expression of *SmeMYB*s based on transcriptome sequencing of five tissues with different colors: leaf, stem, calyx, flower, and fruit. **(h-1)** Three leaf colors: dark purple (DPL), purple-green (PGL), and green (GL). **(h-2)** Three stem colors: dark purple (DPS), purple-green (PGS), and green (GS). **(h-3)** Three calyx colors: dark purple (DPC), purple-green (PGC), and green (GC). **(h-4)** Three flower colors: dark purple (DPF), purple-green (PGF), and green (GF). **(h-5)** Four fruit colors: black purple (B), purple (P), green (G), and white (W). **(h-6)** Expression levels of six differentially expressed *SmeMYB*s in different tissues of HQ-1315. Data were normalized to construct the heatmap. Green and red indicate lower and higher transcript abundance, respectively.

AUTHOR CONTRIBUTIONS

Q.W. and C.B. conceived and designed the research. W.W., T.H., and H.H. prepared the plant materials and managed the fieldwork. J.W., Y.Y., N.H., and H.P. performed sampling, sequencing, and data analysis. Y.W. performed the cytological chromosome analysis. Q.W., W.W., and Y.W. contributed to project discussions. Q.W., Y.W., and W.W. wrote the manuscript, and Q.W., C.B., and Y.W. revised the manuscript.

SUPPLEMENTAL INFORMATION

Supplemental information is available at *Plant Communications Online*.

Received: June 4, 2025
Revised: August 2, 2025
Accepted: September 22, 2025
Published: September 23, 2025

Qingzhen Wei[1,3], Wuhong Wang[1,3], Yunzhu Wang[2,3], Jiaqi Ai[1], Tianhua Hu[1], Haijiao Hu[1], Jinglei Wang[1], Yaqin Yan[1], Hongtao Pang[1], Na Hu[1] and Chonglai Bao[1,]*

[1]The Institute of Vegetables, Zhejiang Academy of Agricultural Sciences, Hangzhou 310021, China
[2]Key Laboratory for Quality and Safety of Agro-Products, Zhejiang Academy of Agricultural Sciences, Hangzhou 310021, China
[3]These authors contributed equally to this article.
***Correspondence: Chonglai Bao (baocl@zaas.ac.cn)**
https://doi.org/10.1016/j.xplc.2025.101533

REFERENCES

Barchi, L., Pietrella, M., Venturini, L., Minio, A., Toppino, L., Acquadro, A., Andolfo, G., Aprea, G., Avanzato, C., Bassolino, L., et al. (2019). A chromosome-anchored eggplant genome sequence reveals key events in Solanaceae evolution. Sci. Rep. **9**:11769.

Barchi, L., Rabanus-Wallace, M.T., Prohens, J., Toppino, L., Padmarasu, S., Portis, E., Rotino, G.L., Stein, N., Lanteri, S., and Giuliano, G. (2021). Improved genome assembly and pan-genome provide key insights into eggplant domestication and breeding. Plant J. **107**:579–596.

Dubos, C., Stracke, R., Grotewold, E., Weisshaar, B., Martin, C., and Lepiniec, L. (2010). MYB transcription factors in *Arabidopsis*. Trends Plant Sci. **15**:573–581.

Fang, H., Wu, J., Xie, L., Li, Y., Huang, J., Yan, X., He, X., Deng, W., Chen, J., Ji, Y., et al. (2025). Telomere-to-telomere genome assembly of eggplant (*Solanum melongena* L.) promotes gene fine localization of the green stripe (GS) in pericarp. Int. J. Biol. Macromol. **284**:138094.

Hirakawa, H., Shirasawa, K., Miyatake, K., Nunome, T., Negoro, S., Ohyama, A., Yamaguchi, H., Sato, S., Isobe, S., Tabata, S., et al. (2014). Draft genome sequence of eggplant (*Solanum melongena* L.): the representative solanum species indigenous to the old world. DNA Res. **21**:649–660.

Jiang, C.K., and Rao, G.Y. (2020). Insights into the diversification and evolution of R2R3-MYB transcription factors in plants. Plant Physiol. **183**:637–655.

Li, D., Qian, J., Li, W., Yu, N., Gan, G., Jiang, Y., Li, W., Liang, X., Chen, R., Mo, Y., et al. (2021). A high-quality genome assembly of the eggplant provides insights into the molecular basis of disease resistance and chlorogenic acid synthesis. Mol. Ecol. Resour. **21**:1274–1286.

Li, J., Jiang, S., Yang, G., Xu, Y., Li, L., and Yang, F. (2023). RNA-sequencing analysis reveals novel genes involved in the different peel color formation in eggplant. Hortic. Res. **10**:181.

Song, B., Song, Y., Fu, Y., Kizito, E.B., Kamenya, S.N., Kabod, P.N., Liu, H., Muthemba, S., Kariba, R., Njuguna, J., et al. (2019). Draft genome sequence of *Solanum* aethiopicum provides insights into disease resistance, drought tolerance, and the evolution of the genome. GigaScience **8**:giz115.

Wei, Q., Wang, J., Wang, W., Hu, T., Hu, H., and Bao, C. (2020). A high-quality chromosome-level genome assembly reveals genetics for important traits in eggplant. Hortic. Res. **7**:153.

Zhang, H., Chen, H., Tan, J., Huang, S., Chen, X., Dong, H., Zhang, R., Wang, Y., Wang, B., Xiao, X., et al. (2023). The chromosome-scale reference genome and transcriptome analysis of *Solanum torvum* provides insights into resistance to root-knot nematodes. Front. Plant Sci. **14**:1210513.

Plant Communications, Volume *6*

Supplemental information

A complete telomere-to-telomere genome assembly of *Solanum melongena* uncovers key regulators in pan-tissue anthocyanin biosynthesis

Qingzhen Wei, Wuhong Wang, Yunzhu Wang, Jiaqi Ai, Tianhua Hu, Haijiao Hu, Jinglei Wang, Yaqin Yan, Hongtao Pang, Na Hu, and Chonglai Bao

Supplemental Figures

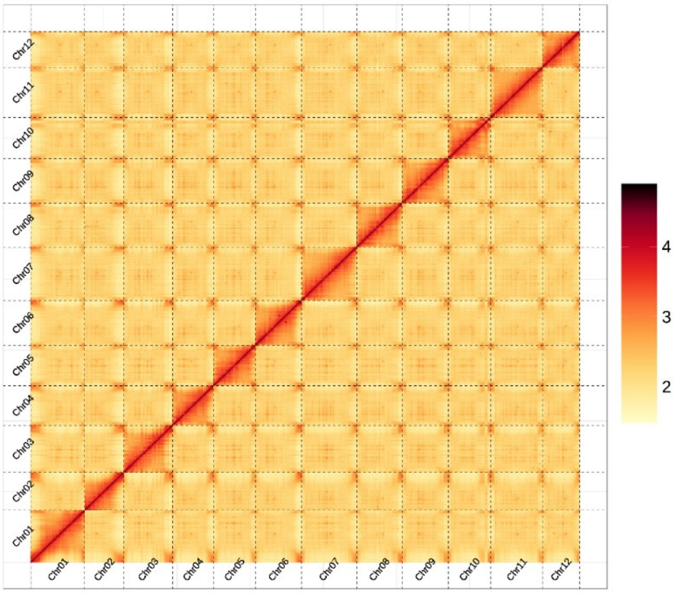

Figure S1 Hi-C chromatin interaction map of the Smel HQ v2.0 assembly.

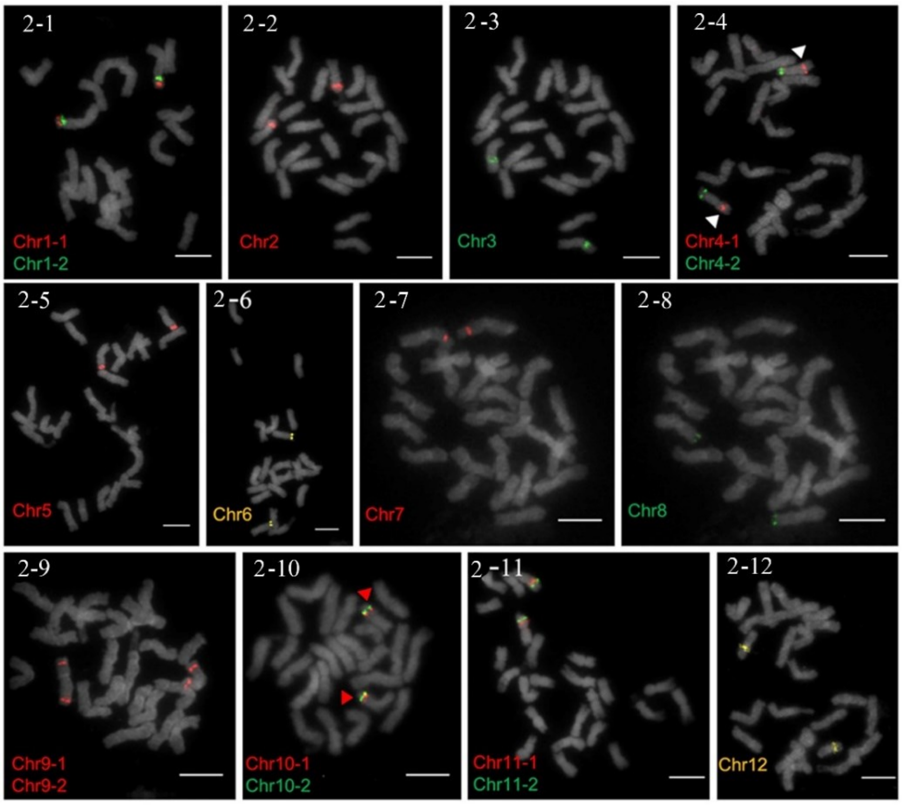

Figure S2 Distribution of the 19 oligo probes on mitosis metaphase chromosomes designed for the identification of 12 eggplant chromosomes and inversion validation. 2-1 to 2-12 showed FISH signals of the oligo probe pools on chromosomes 1-12, respectively.

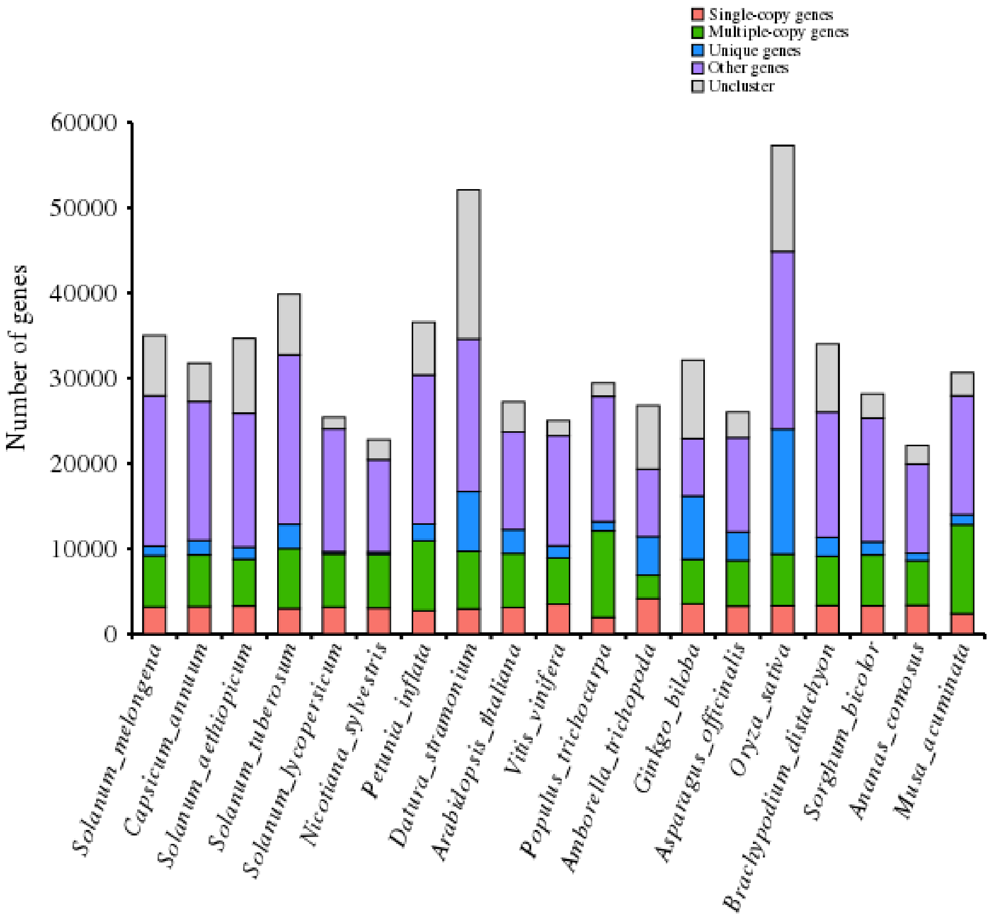

Figure S3 The distribution of genes in different species.

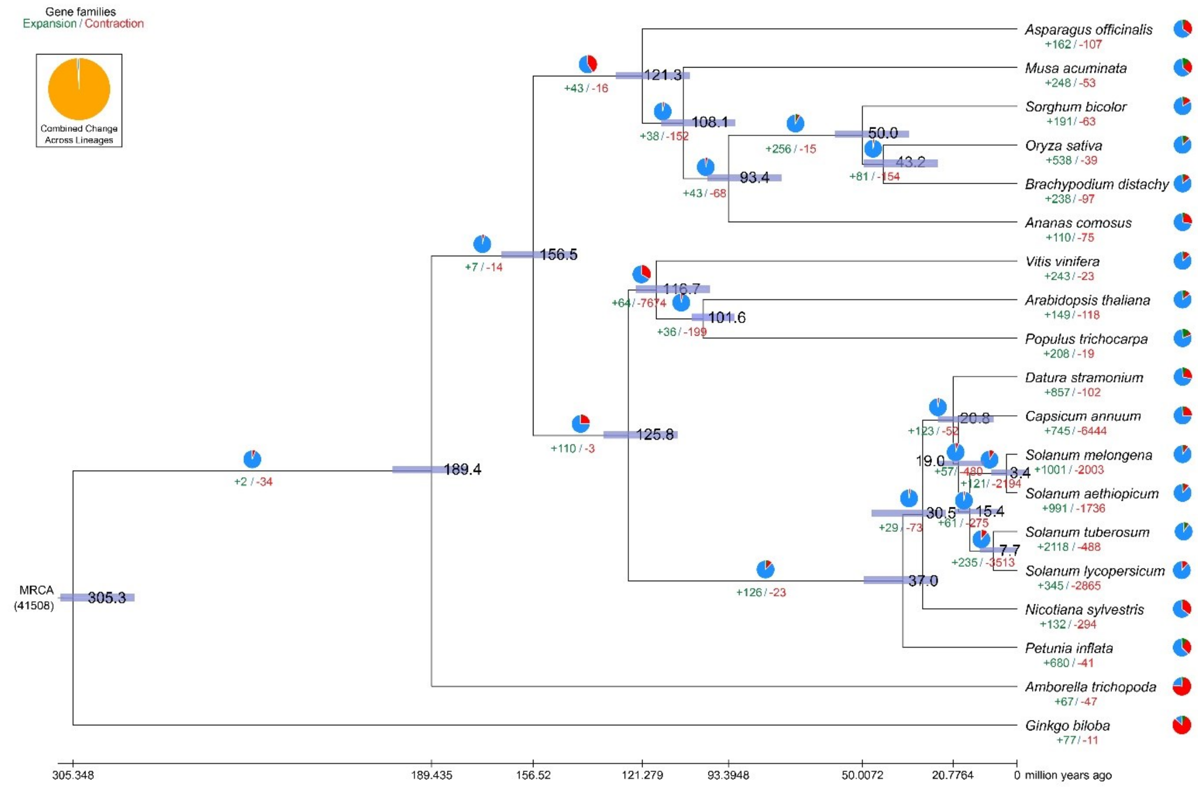

Figure S4 Phylogenetic relationship, divergence time, and expansion and contraction of gene families between S. melongena and 18 plant species. MRCA: most recent common ancestor. The divergence times (in million years) are indicated at each node in blue rectangles. The numbers of expanded gene families are indicated with green numbers, and contracted gene families are indicated with red. represent the conserved gene families are represented by blue portions in the pie charts, while the gene families of MRCAs that expanded or contracted during late divergence are represented by orange proportions.

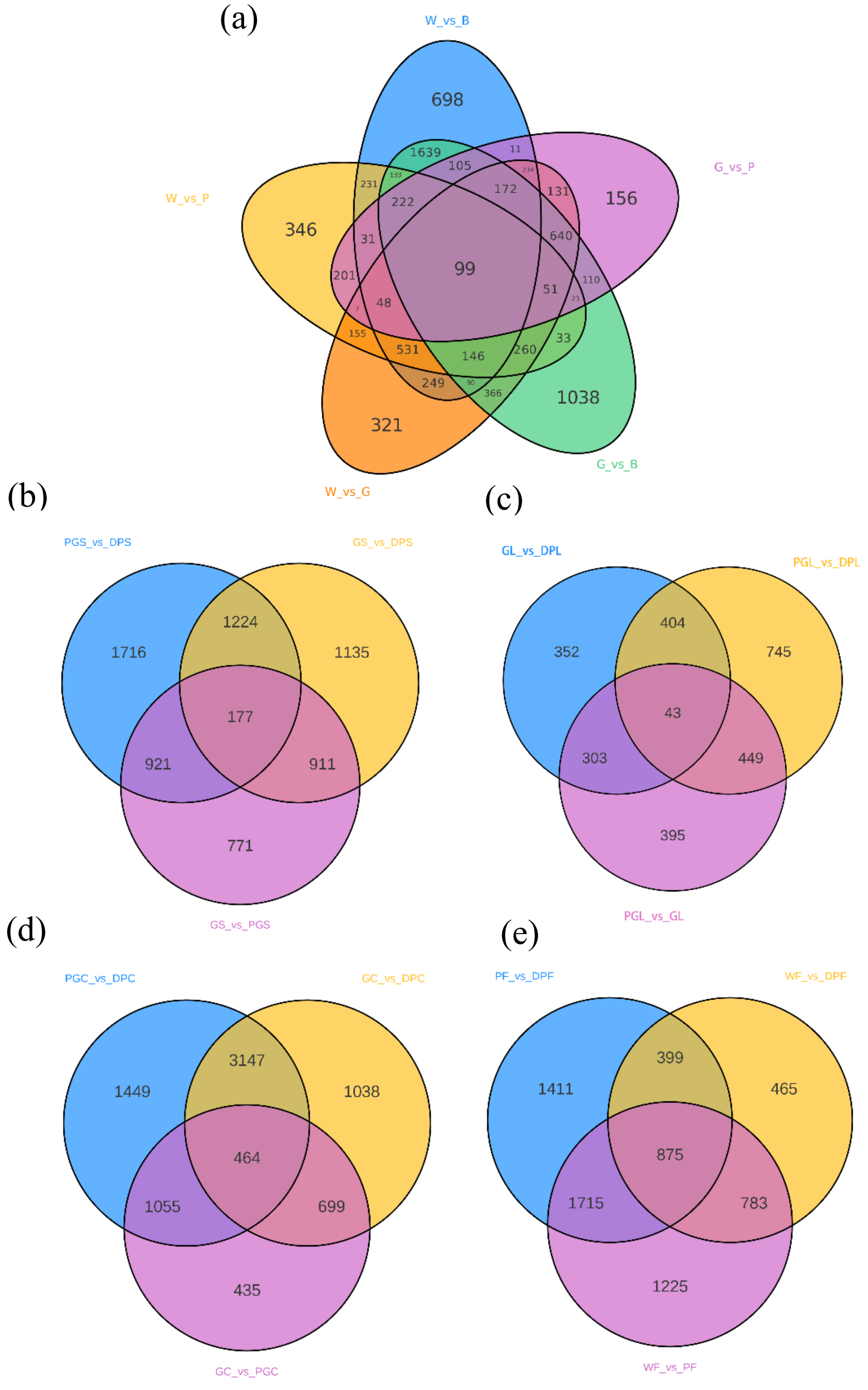

Figure S5 Venn diagram of the common and unique gene in different tissue. (a) peel; (b) stem; (c) young leaf; (d) calyx; (e) flower

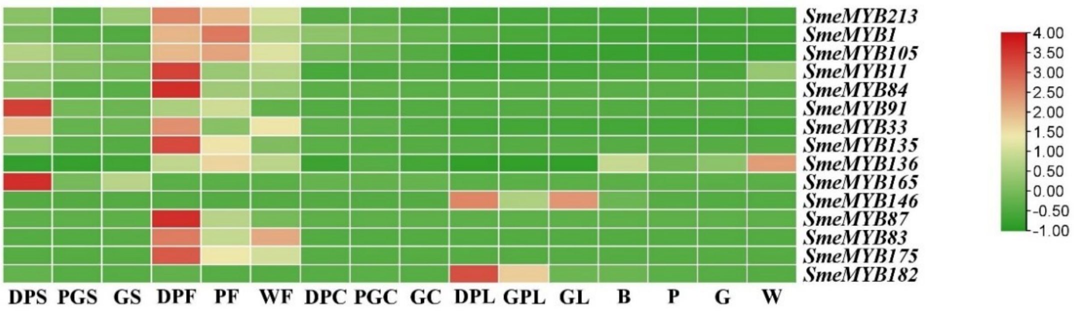

Figure S6 Gene expression of *MYB* transcription factors associated with anthocyanin synthesis in different eggplant tissues. Expression of the *SmeMYBs* in subgroups S4, S5, S6, S7 and S9 that may be associated with anthocyanin synthesis, according to phylogenetic relationships with that of *Arabidopsis*.

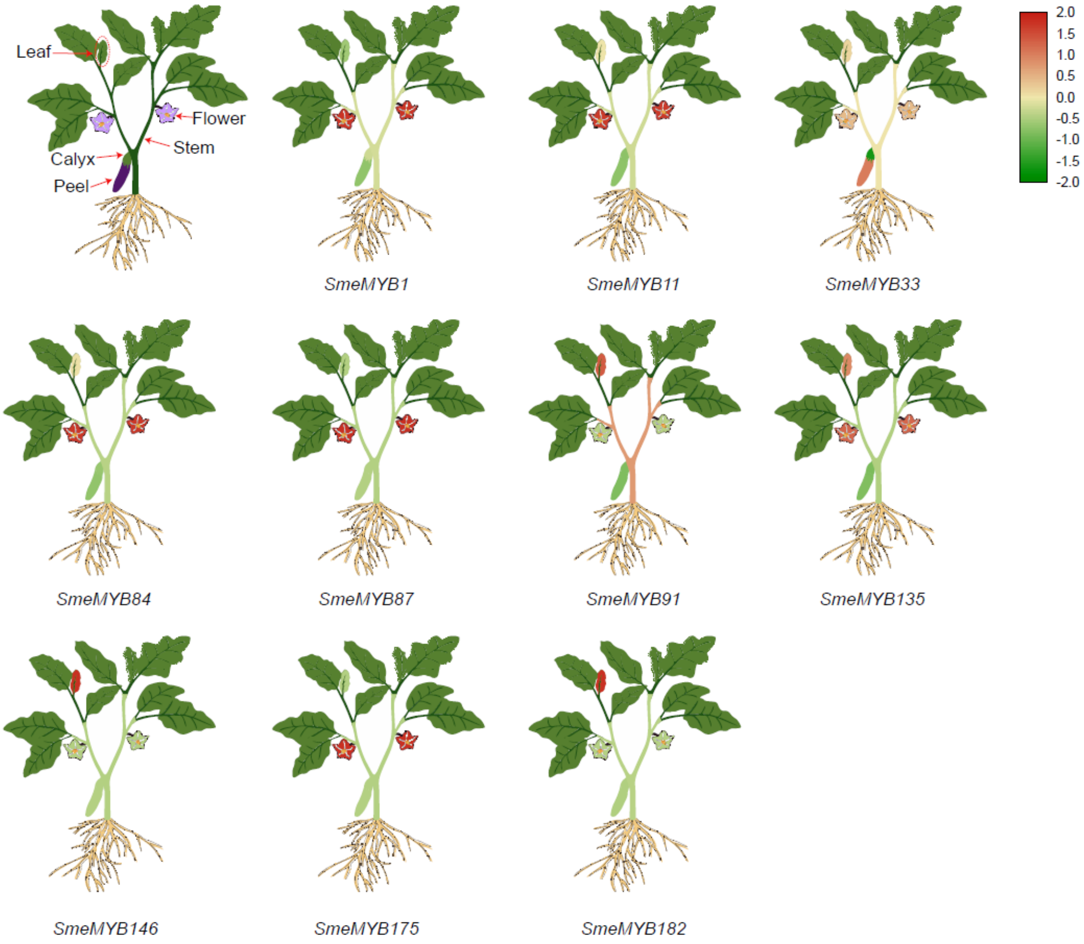

Figure S7 Expression patterns of the 10 differentially expressed *SmeMYBs* in different tissues of HQ-1315. Data were normalized to construct the heatmap. Green indicates low transcript abundance, and red indicates high transcript abundance.
